# Laser and Aesthetic Procedures in Patients with Vitiligo: Patient Selection, Safety, and Practical Clinical Recommendations

**DOI:** 10.3390/jcm15218443

**Published:** 2026-10-29

**Authors:** Amin Elmubarak, Musaab Ahmed

**Affiliations:** 1 Global Care Hospital, Abu Dhabi P.O. Box 2239, United Arab Emirates; 2 College of Medicine, Ajman University, Ajman P.O. Box 346, United Arab Emirates

**Keywords:** vitiligo, Koebner phenomenon, laser therapy, cosmetic, aesthetic procedures, patient safety, leukoderma

## Abstract

**Background:** Patients with vitiligo increasingly seek laser and aesthetic procedures. Current vitiligo guidelines primarily address medical interventions, with limited dedicated guidance on the safety of elective laser and cosmetic procedures. **Objective:** To evaluate the safety of laser and aesthetic procedures in patients with vitiligo and to propose practical risk-stratified clinical recommendations. **Methods:** This narrative review searched PubMed/MEDLINE and Embase from inception to 30 July 2026 for literature on vitiligo, disease activity and stability, Koebnerization, procedure-induced leukoderma, and laser and aesthetic procedures. Evidence from relevant original studies, case reports, case series, systematic reviews, and expert consensus documents was critically synthesised. Where direct procedure-specific evidence was unavailable, recommendations were informed by the extent of tissue trauma, epidermal disruption, thermal or chemical injury, inflammatory burden, mechanistic plausibility, and clinical judgement. **Results:** Procedure induced vitiligo appears to be uncommon but may occur even in clinically stable disease. Risk is likely influenced by current disease activity, previous Koebnerization, and type, intensity and depth of procedural trauma. A four-tier, mechanism-based framework, based on available evidence, expert opinion, and mechanistic reasoning rather than validated comparative studies, is proposed, ranging from minimal-risk procedures, such as botulinum toxin injections to high-trauma interventions such as ablative laser resurfacing. Conservative treatment parameters and avoidance of excessive tissue injury are recommended. Vitiligo-specific safety data remain limited for many aesthetic procedures. **Conclusions:** Vitiligo should not be considered an absolute contraindication to laser or aesthetic procedures. Individualised assessment of disease activity, stability, previous Koebnerization, treatment site, and procedural trauma risk may support safer patient selection. Prospective studies and consensus methodology are needed to validate procedure-specific risk estimates and establish standardised safety recommendations.

## 1. Introduction

Vitiligo is an acquired, chronic depigmenting disorder caused by immune-mediated destruction of epidermal melanocytes. Its global prevalence is estimated at 0.4–1%, with rates varying substantially by region and country [1]. Its pathophysiology centres on a CD8+ T-cell-driven autoimmune attack on melanocytes, amplified by the IFN-γ–CXCL9/CXCL10–CXCR3 signalling axis that recruits and sustains autoreactive T cells within the epidermis [2]. Vitiligo is associated with clinically significant depression and anxiety, alongside stigmatisation, sleep disturbance, sexual dysfunction, relationship difficulty, and occupational impairment [3,4]. In a recent case–control study, moderately severe and severe depressive symptoms were more frequent in patients with vitiligo compared to controls [5]. This psychological burden may increase the desire for aesthetic improvement while also heightening vulnerability to the psychological distress associated with new or worsening depigmentation following aesthetic procedures. There is escalating demand among patients with vitiligo for hair removal, skin rejuvenation, injectables, resurfacing, and other aesthetic procedures. This demand is not addressed by the major vitiligo management guidelines, which focus on medical and phototherapeutic treatment rather than elective cosmetic intervention [6,7,8]. Prior single-source commentary has also called for Koebner-phenomenon risk minimization in this population [9]. The only formal consensus specifically addressing procedural safety in this population focuses on lasers and intense pulsed light (IPL) [10], while guidance on other aesthetic procedures remains limited [9]. To our knowledge, no previous review has systematically evaluated the vitiligo-specific evidence for individual modalities across the full spectrum of laser and aesthetic procedures while considering disease activity and stability. This review synthesises the available evidence, highlights evidence gaps, and proposes a working framework based on available evidence, expert opinion, and mechanistic reasoning to support clinical decision-making and guide future research. The synthesis is translated into practical clinical tools (Table 1, Table 2, Table 3 and Table 4).

Vitiligo is classified into (1) non-segmental disease, the predominant type, typically bilateral and more frequently progressive; (2) segmental disease, typically unilateral and often stabilising more rapidly; (3) the rarer mixed form; and (4) unclassified forms [6]. The course of non-segmental vitiligo is unpredictable, and reactivation can occur even after years, so Koebnerization risk is a dynamic rather than a fixed patient characteristic [11].

Vitiligo is associated with several comorbidities, particularly autoimmune and connective tissue diseases, including autoimmune thyroid disease, type 1 diabetes mellitus, and alopecia areata, as well as other metabolic disorders, underscoring the importance of appropriate comorbidity screening [12]. This overlap suggests shared immune mechanisms and is relevant to procedural planning, because patients may have more than one inflammatory skin disease with its own tendency to Koebnerize and may receive concurrent immunomodulatory treatment. The risk of any skin cancer was significantly lower in patients with vitiligo compared to comparators in a large-scale retrospective propensity score-matched study [13]. Depigmentation is not specific to vitiligo. Immune checkpoint inhibitors used in melanoma treatment may induce vitiligo-like depigmentation and poliosis [14]. Similarly, halo (Sutton’s) naevi and regressing melanoma may present with overlapping clinical and histopathological features [15]. Accurate diagnosis is essential before aesthetic procedures to avoid treating an undiagnosed pigmented lesion or misinterpreting treatment-associated depigmentation as vitiligo.

Vitiligo is classically recognised to exhibit true Koebnerization. It occurs provided that injury reaches the dermal epidermal junction and generates sufficient local inflammatory signal [11]. Cutaneous trauma is thought to trigger Koebnerization through oxidative stress and mechanical detachment of marginally adherent melanocytes (melanocytorrhagy). This releases keratinocyte-derived danger signals and autoantigens that recruit and activate autoreactive CD8+ T cells at the injured site [11]. Onset is typically delayed 10–20 days after trauma but has been documented up to months later, creating potential medicolegal exposure when new depigmentation follows an elective cosmetic procedure [11,16]. A personal history of Koebnerization is reported in 21–62% of patients across heterogeneous series [11].

## 2. Methods

**Figure 1:**
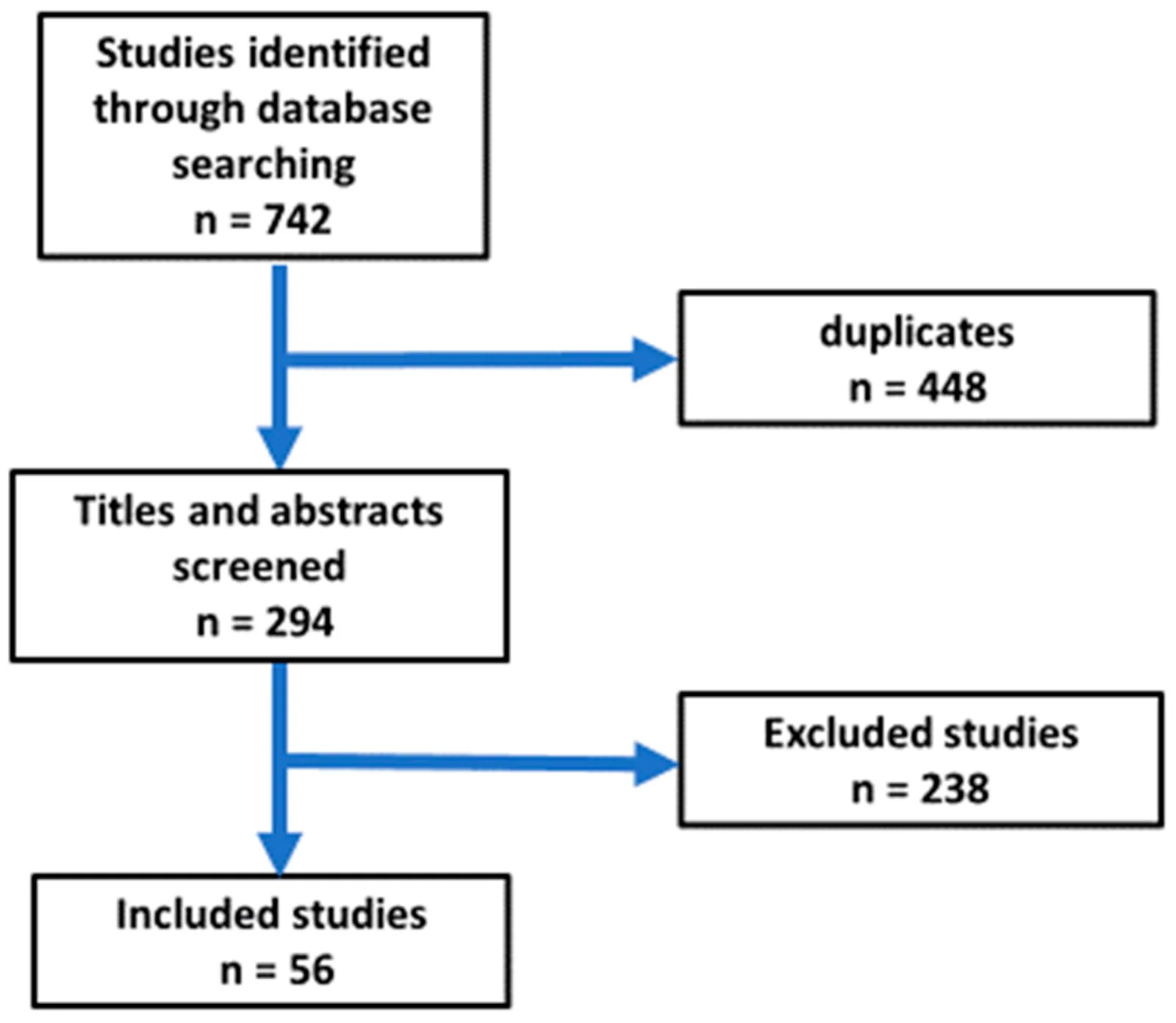
Flowchart of the selection of publications for the narrative review.

This narrative review included a search of the PubMed/MEDLINE and Embase databases for literature addressing laser and aesthetic procedures in patients with vitiligo. The search used combinations of the following terms: vitiligo, laser, aesthetic procedure, risk stratification, and practical clinical recommendation. The literature search was conducted from database inception to 30 July 2026 and was restricted to articles published in English. The search strategy combined terms related to vitiligo with terms related to laser and aesthetic procedures and their safety. Titles and abstracts were screened for relevance, followed by evaluation of the full texts of potentially relevant articles. Relevant information was extracted regarding the type of laser or aesthetic procedure, patient characteristics where available, evidence of procedure-induced vitiligo or Koebnerization, procedural risk, risk stratification, and practical clinical recommendations. The initial search identified 742 records. After removal of duplicate records and screening according to the eligibility criteria, 56 articles were selected for inclusion in the narrative review (Figure 1).

Eligible publications included original research studies, case reports, case series, systematic reviews, and expert consensus documents relevant to laser or aesthetic procedures in patients with vitiligo. All retrieved records were transferred to EndNote for reference management and removal of duplicates. Titles and abstracts were subsequently evaluated, and potentially relevant articles were assessed according to the predefined eligibility criteria. The included literature was critically analysed and narratively synthesised, with particular emphasis on patient selection, disease activity and stability, Koebnerization risk, procedural trauma, risk stratification, and practical measures for improving procedural safety.

## 3. Assessment of Disease Activity, Stability, and Individual Risk

Distinguishing active from stable disease is a key determinant of procedural risk and should be central to patient selection [11,17,18]. A history of new lesion appearance or enlargement of existing lesions indicates activity. Wood’s lamp examination can be used to recognise clinical signs of activity. It includes ill-defined or hypochromic borders, a trichrome pattern, and confetti-like depigmentation [17]. Dermoscopy activity signs include trichrome pattern, reverse pigment network, white nacreous globules, starburst appearance, comet-tail streaks, micro-Koebner phenomenon, perilesional polka dots, and perifollicular depigmentation [18].

Stability refers to the absence of new or enlarging lesions for at least 12 months [6,7]. In one e-Delphi consensus, an international panel agreed that laser/IPL treatment should be withheld unless the patient’s vitiligo has been stable for at least one year and no clinical activity signs are present [10]. Vitiligo stability reduces but does not eliminate risk, as shown in a survey study of laser/IPL-induced leukoderma [19]. A personal Koebnerization history independently predicts future reactivation and greater disease extent. It should be regarded as an independent risk escalator irrespective of current stability [11,20]. Vitiligo activity is inherently dynamic, so risk assessment should be performed at each visit [6].

Aesthetic procedures in patients with vitiligo carry a risk of inducing vitiligo de novo or exacerbating existing disease. Despite this well-characterised risk, dedicated procedure-specific guidance for aesthetic practice in patients with vitiligo remains limited in the literature, particularly beyond laser interventions [10,19]. This places clinicians in a genuine dilemma: either withholding aesthetic care that adds more burden to cosmetic concern, or proceeding with treatment that exposes patients to a small but real risk of procedure-induced depigmentation. This review critically evaluates the safety of laser and aesthetic procedures in patients with vitiligo and provides practical, evidence-based, risk-stratified clinical recommendations for dermatologists and aesthetic practitioners.

The risk of Koebnerization is influenced by the depth, extent, and inflammatory intensity of procedural trauma [11]. We adopt a trauma-based, four-tier risk framework, based on available evidence, expert opinion, and mechanistic reasoning rather than validated comparative studies: minimal risk (fine-needle, minimal-point procedures with no intended inflammatory response, e.g., botulinum toxin), low risk (limited photothermal injury or repetitive fine-needle trauma, e.g., conservative laser hair removal), moderate risk (controlled, partial-thickness or limited-area injury, e.g., non-ablative lasers), and high risk (full-thickness or inflammatory broad-area injury, e.g., ablative resurfacing). This framework is summarised in Table 1.

De novo vitiligo developing after a laser treatment should be considered an absolute contraindication to continuing further laser treatment of the skin [10]. Relative contraindications include stability of less than one year duration and extensive body surface area involvement [9].

Before starting a laser or aesthetic procedure for vitiligo patients, assess benefit versus risk through a shared decision-making process [10]. Assess activity and stability status and elicit Koebnerization history. Examine procedural site risk and skin beyond the intended treatment field for signs of vitiligo. Review concurrent vitiligo therapy (topical corticosteroids, calcineurin inhibitors, JAK inhibitors, phototherapy, or systemic immunomodulators). Many patients use NB-UVB phototherapy or 308 nm excimer light, often with topical calcineurin inhibitors, and combination regimens have been compared in several meta-analyses and trials [21,22,23,24]. Procedure timing should be coordinated with the treating physician to avoid cumulative inflammatory or thermal injury at the same site. Perform a baseline full-skin examination with dermoscopy to document existing lesions and to identify pigmented lesions that are atypical, regressing, or surrounded by a depigmented halo. Review the medication history, including immune checkpoint inhibitors and other agents that may induce depigmentation, because new depigmentation in these patients may not be procedure-induced. It is important to assess the psychosocial distress that would result if new depigmentation occurred [6]. The pre-procedural assessment is summarised in Table 2.

**Figure 2:**
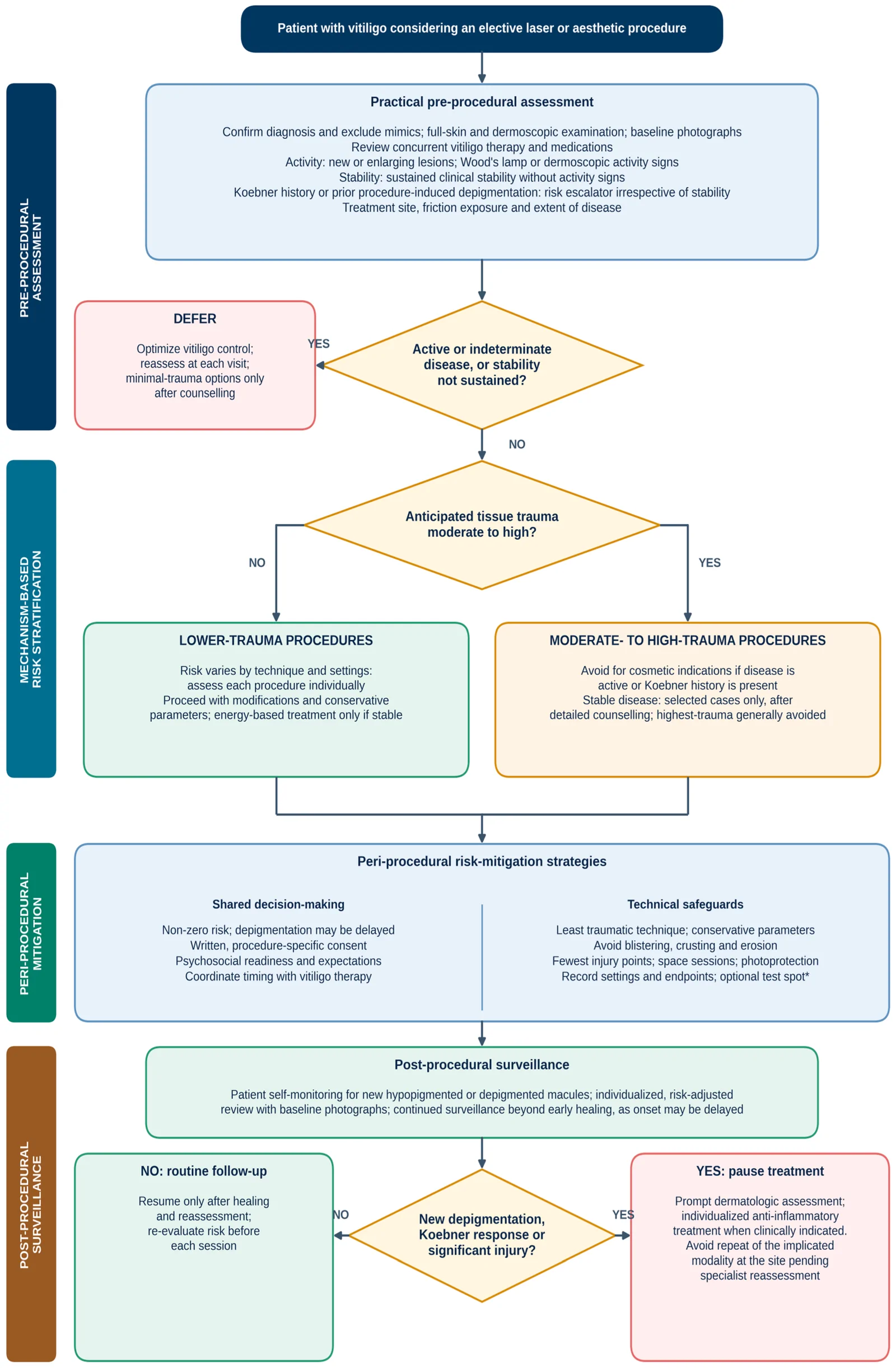
Practical algorithm for patient selection, procedural safety, and follow-up of laser and aesthetic procedures in patients with vitiligo.

In moderate- or high-risk procedures, perform a test spot treatment before therapy to establish appropriate individual settings. Avoid post-treatment endpoints of blistering, crusting, or erosion, as these increase Koebnerization risk [10]. Minimise injection points and favour microcannula over needle technique [25]. Adequate spacing between sessions should be allowed to permit full inflammatory resolution. Regular follow-up is recommended. Patients should be instructed to self-monitor and report any new hypopigmentation. Initiate anti-inflammatory therapy with topical corticosteroids or topical calcineurin inhibitors within one week if post-treatment complications (blistering, crusting, ulceration, or erosion) occur or new depigmentation is identified. Phototherapy can be considered if there is no response to topical treatment [10,25,26]. Peri-procedural risk-mitigation strategies and post-procedural surveillance are summarised in Table 3 and Table 4, respectively, and the overall pathway from assessment to follow-up is shown in Figure 2.

## 4. Laser and Aesthetic Procedures

Botulinum toxin: It is indicated for dynamic rhytid reduction, masseter reduction, and hyperhidrosis. A meeting abstract reported depigmented patches at the injection sites in a patient with cerebral palsy who received multiple high-dose botulinum toxin injections [27]. However, one study of 26 patients with facial spasm found no evidence of periocular depigmentation after botulinum toxin injection [28]. No case reports of vitiligo following botulinum toxin at the regular doses used for wrinkle treatment were identified. This suggests a favourable safety profile but is not entirely risk free.

Laser hair removal: Long-pulsed Nd:YAG, alexandrite, and diode lasers achieve hair reduction through the principle of selective photothermolysis. The optical energy is preferentially absorbed by melanin containing targets to produce localised thermal injury [29]. The risk of inducing vitiligo even under ideal circumstances must be discussed with the patient before treatment. It is better to be withheld unless disease has been stable for at least one year with no activity signs present [10]. A further consideration that may affect the decision to perform the procedure is destruction of hair follicles, which may reduce the potential for repigmentation from hair-follicle stem cells. A multinational cross-sectional expert survey reported a frequency of laser-induced vitiligo of 0.27%, most commonly following hair-removal treatment (46.7% of affected cases). Most cases in this survey occurred in patients without a known prior vitiligo diagnosis [19]. This figure should be interpreted cautiously: it is a survey-based estimate, not prospective incidence data, and it is subject to recall, reporting, and selection bias; it cannot be generalised to specific devices, procedures, or patient populations. Individual case reports corroborate Koebnerization following alexandrite hair-removal laser [30]. Evidence for laser hair removal in patients with vitiligo is limited to case reports, a survey, and consensus guidance. With appropriate parameters and caution, pigmentary adverse events have been reported infrequently [10,19,29,30,31].

Q-switched and picosecond lasers: These lasers produce predominantly photoacoustic and photomechanical effects on melanin-containing structures [29]. In vitiligo patients, they are used for deliberate depigmentation treatment in refractory universalis cases. Patients with active disease at the time of treatment achieved significantly greater depigmentation than those with stable disease [32]. In cosmetic practice, these lasers are used for pigmented lesion or tattoo removal. These lasers target and destroy epidermal melanin and also initiate an inflammatory process that leads to subsequent depigmentation [33]. They should be used with extra caution in patients with vitiligo. However, the available evidence does not support a universal contraindication [34].

Ablative resurfacing lasers (CO_2_, Er:YAG): Fully ablative CO_2_ (10,600 nm) and Er:YAG (2940 nm) lasers vaporise the epidermis and variable depths of the dermis. Fractional ablative CO_2_ laser can be used as a narrowband UVB adjunct in refractory, stable disease. One meta-analysis found significantly higher rates of repigmentation than narrowband UVB alone [35]. A second heterogeneous meta-analysis found only a non-significant trend [36]. A subsequent randomised controlled trial corroborated significantly greater repigmentation with fractional CO_2_ laser plus NB-UVB than with NB-UVB monotherapy alone [37]. Fractional CO_2_ laser has also been combined with topical tacrolimus, calcipotriol, or NB-UVB in stable non-segmental vitiligo [38]. These lasers are also effective in vitiligo treatment when combined with 5-FU according to an open uncontrolled efficacy study in acral vitiligo [39]. They can also be used therapeutically for recipient-site preparation before melanocyte–keratinocyte transplantation [40]. Ablative fractional CO_2_ and Er:YAG lasers are used aesthetically for photoaging and scar resurfacing. Given the full-thickness injury involved, these lasers carry a higher risk of de novo vitiligo or reactivation of existing vitiligo lesions [34]. These lasers are better avoided in patients with vitiligo when used for cosmetics reasons. If treatment is deemed necessary, it should be restricted to stable, localised disease with detailed consent.

Vascular lasers: Pulsed-dye-laser-induced vitiligo has rarely been reported [41]. Because these laser settings generally operate at lower fluences with a reduced likelihood of overt epidermal injury, the procedural risk is plausibly lower still. Conservative fluence principles should be applied to minimise unnecessary epidermal injury and avoid excessive treatment endpoints such as blistering or crusting [19,41].

Microneedling (conventional): Percutaneous microneedling, performed using a dermaroller, dermastamp, or motorised dermapen, creates controlled cutaneous microinjuries and is widely used for cosmetic indications, including acne scarring, fine lines, and transdermal delivery of therapeutic agents. In vitiligo, microneedling has been investigated as an adjunctive treatment for localised, stable disease, usually in combination with topical agents or phototherapy [42]. Additional approaches include combinations with topical 5-fluorouracil [43] and experimental microneedle-based drug-delivery systems [44]. However, the available evidence should be interpreted cautiously because studies are generally small, heterogeneous, and of limited duration, and were not designed to assess the risk of disease reactivation. A dedicated review identifies the Koebner phenomenon as a recognised adverse effect and active vitiligo as a contraindication [45]. Evidence supporting therapeutic microneedling in selected patients with stable vitiligo does not establish its safety for cosmetic indications. Accordingly, cosmetic microneedling cannot currently be recommended for patients with vitiligo, particularly those with active disease or a history of the Koebner phenomenon. If considered, careful patient selection, avoidance of active or previously Koebnerized sites, and explicit counselling regarding the risk of new or expanding depigmentation are essential.

Radiofrequency (RF) microneedling: RF microneedling combines mechanical injury with thermal energy delivered through needle tips, generating localised dermal heat zones and stimulating neocollagenesis. It is used to treat rhytids, skin laxity, and scarring [46]. No controlled studies or case series evaluating its safety in patients with vitiligo were identified. The combined mechanical and thermal injury may theoretically increase the risk of the Koebner phenomenon compared with conventional microneedling, although this has not been established clinically. Accordingly, RF microneedling should be avoided in patients with active vitiligo. In patients with stable disease, its use should generally be discouraged and considered only in exceptional circumstances following careful risk–benefit assessment and explicit counselling regarding the uncertain risk of procedure-induced depigmentation.

Chemical peels: They are used for photoaging and acne scarring. Chemical peels induce controlled chemical injury and epidermal to papillary dermal necrosis that triggers re-epithelialization [47]. In vitiligo patients, peels can be used as an adjunct repigmentation therapy and for depigmentation of residual pigmented skin in extensive disease [48,49]. Deeper phenol peeling has historically also been used as a deliberate depigmentation technique in universal vitiligo because it ablates melanocytes [49]. The use of chemical peels in cosmetic practice is controversial. Superficial peels are reasonable in stable disease, reserving deep peeling for cases where depigmentation is the deliberate goal.

Dermabrasion: Motorised dermabrasion mechanically ablates the epidermis and superficial dermis. It can be used as a method of recipient-site preparation in vitiligo surgery, showing comparable or superior repigmentation to Er:YAG laser and short-pulsed CO_2_ laser in comparative studies [40]. A systematic review confirms an important adjunctive role for phototherapy in optimising repigmentation following surgical or ablative recipient-site preparation in vitiligo [50]. Given the full-thickness nature of the injury, this procedure is high risk and should be avoided, or restricted to stable, localised disease under strict supervision [11].

Dermal fillers: Unlike several other autoimmune conditions that warrant individualised risk assessment, vitiligo is not currently listed among recognised filler contraindications [51]. Injectable fillers such as hyaluronic acid (HA) are used to restore volume and have less intrinsic inflammatory stimulus. Other fillers (such as calcium hydroxyapatite, poly-L-lactic acid, and polycaprolactone) induce a controlled foreign body inflammatory response to stimulate neocollagenesis. A rare case report describes a woman who developed vitiligo two weeks after receiving a polycaprolactone-based collagen-stimulator filler [26]. The microcannula technique is preferred and needle passes should be minimised.

Platelet-rich plasma (PRP), exosomes, polynucleotides, and skin boosters (mesotherapy): These products are delivered via mesotherapy intradermal microinjection to stimulate dermal regeneration, facial rejuvenation, and skin quality improvement [52]. A case report describes non-segmental facial vitiligo developing after the third PRP session, attributed to repetitive microneedle trauma rather than to PRP itself [25]. No dedicated data exist for exosomes, polynucleotides, or skin boosters, despite their increasing use with multipoint intradermal injection techniques. These modalities are regenerative and generally well tolerated in the broader cosmetic population, although their safety in patients with vitiligo remains uncertain. In stable disease, conservative injection techniques that minimise unnecessary tissue trauma and the number of punctures per session are therefore prudent [25,32].

Emerging and understudied procedures.: High-intensity focused ultrasound (HIFU) delivers focused acoustic energy to generate discrete thermal coagulation points for non-invasive skin tightening. Available HIFU literature reports an acceptable safety profile without a clear signal for dyspigmentation or Koebnerization. However, epidermal-sparing energy delivery does not eliminate the theoretical risk of dermal thermal injury [53]. A larger, more recent systematic review of HIFU in aesthetic practice corroborated this generally favourable safety profile, reporting predominantly mild and transient adverse events [54]. Thread lifting combines cannula insertion trauma with an intentional inflammatory response (biostimulating threads) analogous to biostimulator fillers [26]. LED/photobiomodulation therapy delivers non-ionising, low-level light without epidermal disruption. Limited vitiligo-specific evidence suggests minimal adverse events, although standardised protocols and long-term safety data remain lacking [55].

## 5. Discussion

Koebnerization risk may increase with trauma depth, extent, and inflammatory intensity. It is modulated by disease activity at the time of treatment, regardless of whether the injurious stimulus is mechanical, thermal, chemical, or biologically inflammatory [11]. This tiering reflects expert-informed mechanistic reasoning rather than validated procedure-specific incidence data and should therefore be regarded as a working clinical framework pending prospective outcome studies. The Koebner phenomenon may be related to the inflammatory pathways that drive vitiligo. The IFN-γ–CXCL9/CXCL10–CXCR3 axis is an important pathway in the recruitment of autoreactive CD8^+^ T cells in vitiligo, while trauma can trigger new lesions through the Koebner phenomenon. Whether trauma directly activates this specific pathway remains to be established [2,11]. This principle is codified for laser treatment specifically in a consensus guidance document. Other procedures still lack comparable formal guidance. Recommendations for disease stability, absence of activity signs, and avoidance of overtly injurious parameters may provide a framework for future consensus recommendations on aesthetic procedures, pending controlled trial data [10]. Available evidence suggests that laser-induced vitiligo is uncommon, with only 30 cases reported among 11,300 patients (0.27%) in an international survey of vitiligo experts, although this survey-based figure is not a prospective incidence estimate and may over or underestimate true risk (see Section 5.2). Reported cases were frequently associated with treatment-related skin injury, supporting a cautious approach to laser and aesthetic procedures rather than routine avoidance of aesthetic laser treatment in all patients with vitiligo [19]. The risk of Koebnerization means that skin-penetrating aesthetic procedures cannot be considered risk-free [25,26].

### 5.1. Mechanistic Considerations

Vitiligo is driven by an autoimmune CD8^+^ T-cell response against melanocytes, sustained by IFN-γ signalling and the CXCL9/CXCL10–CXCR3 axis [2,56]. Skin injury may act on this primed background. Mechanical or thermal damage can induce oxidative stress and detachment of poorly adherent melanocytes and can release keratinocyte-derived danger signals and melanocyte autoantigens [11]. At the injured site, this inflammation may recruit or reactivate autoreactive T cells, resulting in a Koebner response. This mechanism could explain why risk may vary with the depth and extent of tissue injury and the intensity of the resulting inflammation, as well as why procedures may pose greater risks during active disease. However, direct evidence that specific aesthetic procedures activate the IFN-γ–CXCL9/CXCL10 axis in human skin is lacking, and this model remains inferential [2,11]. The delay between injury and visible depigmentation (typically 10–20 days, occasionally months) further complicates causal attribution [11,16].

### 5.2. Possible Underreporting of Procedure-Induced Vitiligo

Several factors probably lead to underestimation. First, the evidence consists mainly of case reports, small series, and expert surveys, which are susceptible to publication, recall, and selection bias [19,31]. Second, delayed onset makes the link with a procedure hard to recognise for patients and practitioners, and patients may consult a different clinician than the one who performed the procedure [11,16]. Third, procedures performed in aesthetic settings outside dermatology are less likely to be recorded or published, and no registry exists. Fourth, in the survey that provides the only available estimate, most cases occurred in patients without a prior vitiligo diagnosis [19]. This finding raises the possibility that procedures may unmask subclinical disease or coincide with spontaneous disease onset; without baseline documentation, these explanations cannot be distinguished. Mild or transient depigmentation may also remain unreported. Conversely, a temporal association does not prove causation, and coincidental onset may be over-attributed to a procedure. Reported frequencies should therefore be interpreted cautiously. Baseline photographic documentation, standardised definitions, and prospective registries would allow more reliable estimates.

### 5.3. Evidence Gaps and Future Research Priorities

Genuine uncertainty remains regarding the optimal duration of disease stability before aesthetic procedures, as current thresholds are largely extrapolated from surgical vitiligo literature and have not been specifically validated for aesthetic indications [6]. Whether peri-procedural anti-inflammatory treatment reduces the risk of Koebnerization is also inadequately studied [11,20]. The interaction between contemporary vitiligo therapies and procedural risk warrants further investigation. JAK inhibitors modulate the IFN-γ–JAK/STAT–CXCL9/CXCL10 axis implicated in vitiligo pathogenesis and CD8^+^ T-cell recruitment [2,7], but whether this translates into protection against procedure-induced Koebnerization remains unknown.

Evidence for aesthetic procedures in patients with vitiligo remains limited, consisting predominantly of case reports, small series, and expert consensus rather than controlled studies [10,19,31]. Future research should prioritise prospective studies using standardised definitions of disease stability and Koebnerization and evaluating predictors of procedure-related disease activation. A Delphi consensus could complement the limited empirical evidence by establishing procedure-specific recommendations for patient selection, risk stratification, and risk-mitigation strategies. Development and validation of a standardised Koebnerization risk-assessment tool should further support individualised decision-making and procedural safety.

### 5.4. Study Limitations

This review has several limitations. First, it was conducted as a narrative review rather than a systematic review or meta-analysis, so we did not perform a formal quantitative synthesis or assess publication bias. Second, evidence on the safety of many laser and aesthetic procedures in patients with vitiligo remains limited. Most available data come from case reports, case series, observational studies, and expert consensus. As a result, the true incidence of procedure-induced vitiligo or Koebnerization, as well as the strength of the association between specific procedures and disease activation, remains uncertain.

In addition, the procedures vary considerably in their treatment parameters, depth, intensity, anatomical site, operator technique, and patient-related factors. This heterogeneity makes direct comparisons of procedural risks difficult. Finally, the proposed four-tier risk-stratification framework is based on the available clinical evidence, expert opinion, and mechanistic considerations. It has not yet been prospectively validated as a quantitative risk-prediction model. Therefore, the categories should be viewed as a practical clinical guide rather than as definitive estimates of procedure-specific risk. Future prospective studies and formal consensus-based research are needed to validate and further refine these recommendations. Furthermore, only publications in English were included. Relevant studies published in other languages may therefore have been missed, introducing language bias, and the extent of its effect on our findings is unknown.

## 6. Conclusions

Aesthetic and laser procedures need not be categorically withheld from patients with vitiligo. Careful assessment of disease activity, stability, and history of the Koebner phenomenon, combined with risk-based patient selection and procedure-specific technical modifications, may allow selected lower-risk procedures to be performed with an acceptable risk profile. Conversely, moderate- to high-risk procedures should generally be avoided for cosmetic indications in patients with active disease and considered cautiously in those with stable disease following appropriate counselling regarding the potential risk of depigmentation. Substantial evidence gaps remain, particularly for newer injectable and energy-based modalities, highlighting the need for prospective studies to better define their safety in this population.

## Figures and Tables

**Table 1:** Mechanism-based risk stratification of laser and aesthetic procedures in patients with vitiligo.

Risk Tier	Representative Procedures	Potential Mechanism of Koebnerization
Tier 1—Minimal	• Botulinum toxin injections • LED/photobiomodulation therapy	Minimal focal tissue trauma without clinically significant thermal or ablative injury; epidermal integrity is largely preserved
Tier 2—Low	• Hyaluronic acid filler • Platelet-rich plasma (intradermal microinjection) • Polynucleotide/skin-booster injections • Long-pulse Nd:YAG laser hair removal (conservative parameters, adequate cooling) • Pulsed dye and other vascular lasers (conservative parameters)	Limited photothermal injury or repetitive fine-needle trauma with largely preserved epidermal integrity and a relatively limited inflammatory response
Tier 3—Moderate	• Intense pulsed light • Non-ablative fractional laser • Radiofrequency microneedling • Conventional microneedling • Superficial chemical peels • High-intensity focused ultrasound • Low-fluence Q-switched/picosecond laser • Thread lifting	Controlled epidermal and/or dermal injury resulting from thermal, mechanical, or chemically mediated inflammation, with greater potential for inflammatory and wound-healing responses
Tier 4—High	• Ablative fractional CO_2_ or Er:YAG laser resurfacing • Dermabrasion • Medium-deep chemical peels • High-fluence Q-switched or picosecond laser • Surgical scar revision	Extensive epidermal–dermal wounding, substantial barrier disruption, and pronounced inflammatory and wound-healing responses with greater potential for Koebnerization

Footnote: Risk tiers represent an expert-informed, mechanism-based clinical framework based on the extent of procedural trauma, epidermal disruption, thermal injury, inflammatory burden, and biological plausibility of Koebnerization, rather than validated procedure-specific incidence rates. Individual risk may be modified by vitiligo activity and stability, previous Koebner phenomenon, treatment parameters, procedure depth and density, anatomical site, skin phototype, and operator technique. This classification is intended to support clinical decision-making and does not represent a validated quantitative risk model.

**Table 2:** Practical pre-procedural assessment framework for patients with vitiligo undergoing laser or aesthetic procedures.

Confirm Vitiligo Subtype and Document Baseline Extent Photographically.
Assess and classify disease activity as active, stable, or indeterminate.
Confirm adequate disease stability before proceeding to the procedure
Elicit history of Koebnerization, including prior procedure-induced vitiligo.
Classify the planned procedure’s risk tier and confirm eligibility (Table 1).
Inspect the treatment site and surrounding skin for active lesions, previously Koebnerized sites, or areas of repeated friction.
Perform baseline full-skin and dermoscopic examination; review drug history and evaluate suspicious pigmented or halo lesions before treating nearby skin.
Review concurrent vitiligo therapy and coordinate timing with the treating physician.
Assess psychosocial readiness and align patient expectations.
Conduct shared decision-making and obtain written, procedure-specific consent.
Consider a conservative test spot before full-field treatment in moderate- or high-risk (Tier 3–4) procedures.

**Table 3:** Peri-procedural risk-mitigation strategies for laser and aesthetic procedures in patients with vitiligo.

Perform Test Spot Before Procedures.
Use conservative treatment parameters and avoid excessive inflammatory and erosive endpoints.
For injectables, minimise injection points and use the least traumatic technique appropriate for the procedure.
Space treatment sessions to allow full inflammatory resolution.
Maintain strict photoprotection throughout healing.
Avoid treating multiple high-friction or previously Koebnerized sites during same session.
Coordinate timing with concurrent vitiligo therapy.
Document treatment parameters, procedural endpoints, and any immediate adverse reactions.

**Table 4:** Post-procedural surveillance and management of procedure-related depigmentation and Koebnerization risk in patients with vitiligo.

Follow a Risk-Adjusted Monitoring Schedule, with Patient Self-Monitoring Throughout.
Instruct patients to promptly report new hypo- or depigmentation.
Consider early anti-inflammatory treatment when clinically appropriate for significant inflammatory complications or new depigmentation.
Escalate to phototherapy if depigmentation fails to respond to first-line treatment.
Avoid further treatment with the same modality at that site pending reassessment when post-procedure de novo vitiligo occurs.

## Data Availability

No new data were created or analysed in this study. Data sharing is not applicable to this article.

## Abbreviations

CD8+, cluster of differentiation 8-positive; CO_2_, carbon dioxide; Er:YAG, erbium-doped yttrium aluminum garnet; HA, hyaluronic acid; HIFU, high-intensity focused ultrasound; IFN-γ, interferon gamma; IPL, intense pulsed light; JAK, Janus kinase; LED, light-emitting diode; NB-UVB, narrowband ultraviolet B; Nd:YAG, neodymium-doped yttrium aluminum garnet; PRP, platelet-rich plasma; QS, Q-switched; RF, radiofrequency; 5-FU, 5-fluorouracil.

## References

[1] Haulrig M.B., Wolkerstorfer A., Speeckaert R., Egeberg A., Thomsen S.F. (2024). The global epidemiology of vitiligo: A systematic review and meta-analysis of the incidence and prevalence. JEADV Clin. Pract..

[2] Liu H., Wang Y., Le Q., Tong J., Wang H. (2024). The IFN-γ-CXCL9/CXCL10-CXCR3 axis in vitiligo: Pathological mechanism and treatment. Eur. J. Immunol..

[3] Picardo M., Huggins R.H., Jones H., Marino R., Ogunsola M., Seneschal J. (2022). The humanistic burden of vitiligo: A systematic literature review of quality-of-life outcomes. J. Eur. Acad. Dermatol. Venereol..

[4] Ezzedine K., Eleftheriadou V., Jones H., Bibeau K., Kuo F.I., Sturm D., Pandya A.G. (2021). Psychosocial effects of vitiligo: A systematic literature review. Am. J. Clin. Dermatol..

[5] Molla A., Albadrani M. (2025). The psychological burden of vitiligo: Investigating the depressive symptoms in patients with vitiligo: A case–control study. Medicina.

[6] van Geel N., Speeckaert R., Taïeb A., Ezzedine K., Lim H.W., Pandya A.G. (2023). Worldwide expert recommendations for the diagnosis and management of vitiligo: Part 1: Towards a new management algorithm. J. Eur. Acad. Dermatol. Venereol..

[7] Seneschal J., Speeckaert R., Taïeb A., Wolkerstorfer A., Passeron T., Pandya A.G. (2023). Worldwide expert recommendations for the diagnosis and management of vitiligo: Part 2: Specific treatment recommendations. J. Eur. Acad. Dermatol. Venereol..

[8] Eleftheriadou V., Atkar R., Batchelor J., McDonald B., Novakovic L., Patel J.V., Ravenscroft J., Rush E., Shah D., Shah R. (2022). British Association of Dermatologists guidelines for the management of people with vitiligo 2021. Br. J. Dermatol..

[9] AlShams A., Baroud R. (2026). Aesthetic procedures in patients with vitiligo: Evidence-based clinical guidelines for minimizing Koebner phenomenon risk. Clin. Surg..

[10] Post N.F., Rodrigues M.A., Liong-A-Jin C., Lommerts A., Abdallah M., Bae J.M., Bekkenk M., Silva de Castro C.C., Eleftheriadou V., Esmat S.M. (2023). Consensus on the safety and risks of laser and intense pulse light treatments in patients with vitiligo: An e-Delphi study. J. Am. Acad. Dermatol..

[11] van Geel N., Speeckaert R., Taieb A., Picardo M., Böhm M., Gawkrodger D.J., Schallreuter K., Bennett D.C., van der Veen W., Whitton M. (2011). Koebner’s phenomenon in vitiligo: European position paper. Pigment Cell Melanoma Res..

[12] Lee J.H., Ju H.J., Seo J.M., Almurayshid A., Kim G.M., Ezzedine K., Bae J.M. (2023). Comorbidities in patients with vitiligo: A systematic review and meta-analysis. J. Invest. Dermatol..

[13] Nazzal Y., Najeeb M., Dagher J., Bertolini M., Passeron T., Ludwig R.J., Curman P. (2026). Vitiligo and skin cancer risk across racial/ethnic groups: A retrospective propensity score-matched study on 123,179 patients. Eur. J. Cancer.

[14] Smart K., Rodriguez I., Swanson M.S., Berry J.L., Terando A., Peng D.H., Worswick S., In G.K. (2026). Development of poliosis in patients treated with immunotherapy for metastatic cutaneous and non-cutaneous melanoma. Pigment Cell Melanoma Res..

[15] Hlača N., Vičić M., Prpić-Massari L. (2026). Immunological aspects of halo nevus (Sutton’s nevus). Front. Immunol..

[16] Sanchez D.P., Sonthalia S. (2022). Koebner Phenomenon. StatPearls [Internet].

[17] van Geel N., Grine L., De Wispelaere P., Mertens D., Prinsen C.A.C., Speeckaert R. (2019). Clinical visible signs of disease activity in vitiligo: A systematic review and meta-analysis. J. Eur. Acad. Dermatol. Venereol..

[18] Boughaleb S., Soughi M., Sqalli G., Oulehri A., El Baldi M., Douhi Z., Elloudi S., Baybay H., El Rhazi K., Mernissi F.Z. (2025). Dermatoscopic markers of disease activity in vitiligo. JAAD Int..

[19] Post N.F., Van Broekhoven N.X., Lommerts A., Bae J.M., Bekkenk M.W., Silva de Castro C.C., Eleftheriadou V., Esmat S., Ezzedine K., van Geel N. (2023). Expert opinion about laser and intense pulsed light (IPL)-induced leukoderma or vitiligo: A cross-sectional survey study. Arch. Dermatol. Res..

[20] Njoo M.D., Das P.K., Bos J.D., Westerhof W. (1999). Association of the Köbner phenomenon with disease activity and therapeutic responsiveness in vitiligo vulgaris. J. Am. Acad. Dermatol..

[21] Liu Z., Ng W.H., Morriss S., Liu Z.F., Lin L., Xireaili F., Chew C. (2026). Excimer 308-nm light in the combination treatment of vitiligo: A systematic review and network meta-analysis. Pigment Cell Melanoma Res..

[22] Chang H.C., Sung C.W. (2021). Efficacy of combination therapy of narrowband-ultraviolet B phototherapy or excimer laser with topical tacrolimus for vitiligo: An updated systematic review and meta-analysis. Photodermatol. Photoimmunol. Photomed..

[23] Zhu B., Liu C., Zhang L., Wang J., Chen M., Wei Y. (2023). Comparison of NB-UVB combination therapy regimens for vitiligo: A systematic review and network meta-analysis. J. Cosmet. Dermatol..

[24] Yamamoto A., Enomoto Y., Sakurai M., Watanabe O., Kanayama Y., Koike Y., Sakaida T., Morita A. (2026). Efficacy of minimal blistering dose-based versus conventional excimer laser therapy for vitiligo. J. Dermatol..

[25] Ejjiyar M., Sahibi M., El Gueouatri M., Bhihi A., Mahrouch M., Yafi I., El Amrani M.D., Benchamkha Y. (2019). Vitiligo and Koebner phenomenon following platelet-rich plasma injections. Pan Afr. Med. J..

[26] Huang C.H., Ng C.Y. (2022). Vitiligo associated with polycaprolactone-based collagen stimulator filler. JAAD Case Rep..

[27] Halverstam C., Alvarez M. (2011). Post-botulinum toxin depigmentation. J. Am. Acad. Dermatol..

[28] Roehm P.C., Perry J.D., Girkin C.A., Miller N.R. (1999). Prevalence of periocular depigmentation after repeated botulinum toxin A injections in African American patients. J. Neuroophthalmol..

[29] Anderson R.R., Parrish J.A. (1983). Selective photothermolysis: Precise microsurgery by selective absorption of pulsed radiation. Science.

[30] Alkhalifah A. (2021). A case report of vitiligo induced by alexandrite hair removal laser. Case Rep. Dermatol..

[31] Post N.F., Van Broekhoven N.X., Bekkenk M.W., Wolkerstorfer A. (2022). Laser- and intense pulsed light (IPL)-induced vitiligo patches: A systematic review of the literature—Short report. Lasers Med. Sci..

[32] Komen L., Zwertbroek L., Burger S.J., van der Veen J.P., de Rie M.A., Wolkerstorfer A. (2013). Q-switched laser depigmentation in vitiligo, most effective in active disease. Br. J. Dermatol..

[33] Modi K., Mohammad T., Hamzavi I. (2018). Use of the 532-nm Q-switched neodymium-doped yttrium aluminum garnet laser for the treatment of recalcitrant repigmentation in vitiligo. JAAD Case Rep..

[34] Karampinis E., Georgopoulou K.E., Goudouras G., Lianou V., Kampra E., Roussaki Schulze A.V., Zafiriou E. (2024). Laser-induced Koebner-related skin reactions: A clinical overview. Medicina.

[35] Kim W.I., Kim S., Lee S.H., Cho M.K. (2021). The efficacy of fractional carbon dioxide laser combined with narrow-band ultraviolet B phototherapy for non-segmental vitiligo: A systematic review and meta-analysis. Lasers Med. Sci..

[36] Chang H.C., Lin M.H., Tsai H.H. (2020). Efficacy of combination therapy with fractional carbon dioxide laser and ultraviolet B phototherapy for vitiligo: A systematic review and meta-analysis. Aesthetic Surg. J..

[37] Rokni G.R., Malekan M., Azade S., Beyzaee A.M., Heydari K., Fallah S., Kazeminejad A., Zaresharifi S., Gholizadeh N. (2025). Fractional CO_2_ laser combined with narrow-band ultraviolet B (NB-UVB) phototherapy versus NB-UVB monotherapy in vitiligo patients: Prospective, self-controlled, and randomized trial. Lasers Med. Sci..

[38] Bakr R.M., Abdel-Gaber R.M., Tawfik Y.M. (2021). A comparative study on the use of fractional CO_2_ laser with tacrolimus or calcipotriol or narrow band ultraviolet-B in treatment of stable nonsegmental vitiligo. Dermatol. Ther..

[39] Weshahy R.H., Abdel Hamid M.F., Sayed K.S., El Desouky E.D., Ramez S.A. (2022). Efficacy and safety of combined fractional ablative CO_2_ laser and 5-fluorouracil in the treatment of acral vitiligo: An open, uncontrolled study. J. Cosmet. Dermatol..

[40] Post N.F., Ezekwe N., Narayan V.S., Bekkenk M.W., Van Geel N., Hamzavi I., Passeron T., Wolkerstorfer A. (2022). The use of lasers in vitiligo, an overview. J. Eur. Acad. Dermatol. Venereol..

[41] Cui W., Wan M., Miao Y., Chen L., Shao L. (2025). Treatment of spider nevus-induced vitiligo using pulsed dye laser in a child. Lasers Med. Sci..

[42] Salloum A., Bazzi N., Maalouf D., Habre M. (2020). Microneedling in vitiligo: A systematic review. Dermatol. Ther..

[43] Abdulqader N.M.B., Qurtas D.S., Sharaf B.K. (2026). Efficacy of topical 5-fluorouracil combined with microneedling in the treatment of stable vitiligo. J. Coll. Physicians Surg. Pak..

[44] Jiang Z., Zhou Y., Wang B., He C., Zhang J., Wang Q., Deng Z., Li C., Jian Z. (2025). Microneedle patch delivering multifunctional melanin-like nanoparticles for vitiligo remission and repigmentation. ACS Appl. Mater. Interfaces.

[45] Abd El-Maged W.M., Saleh R., Abd-Elghany N.I. (2022). Microneedling in treatment of vitiligo. Sohag Med. J..

[46] Kneiber D., Amin M., Nguyen T.A., Gharavi N.M. (2023). Review of radiofrequency microneedling: History, devices and uses. J. Cosmet. Laser Ther..

[47] El Mofty M., Esmat S., Hunter N., Mashaly H.M., Dorgham D., Shaker O., Ibrahim S. (2017). Effect of different types of therapeutic trauma on vitiligo lesions. Dermatol. Ther..

[48] Nofal A., Fawzy M.M., Alakad R. (2021). Trichloroacetic acid in different concentrations: A promising treatment modality for vitiligo. Dermatol. Surg..

[49] Alomar A., Marrón Hernández M., Bittencourt F. (2021). Residual pigment islands treated with 88% phenol peeling in a woman with universal vitiligo. Actas Dermosifiliogr..

[50] Lommerts J.E., Uitentuis S.E., Bekkenk M.W., de Rie M.A., Wolkerstorfer A. (2018). The role of phototherapy in the surgical treatment of vitiligo: A systematic review. J. Eur. Acad. Dermatol. Venereol..

[51] De Boulle K., Heydenrych I. (2015). Patient factors influencing dermal filler complications: Prevention, assessment, and treatment. Clin. Cosmet. Investig. Dermatol..

[52] Yi K.H., Kang H., Song J.K., Sydorchuk O., Green J.B., Cartier H., Zamin B.Z., Ascher B. (2026). Skin boosters: 2026 updated. J. Cosmet. Dermatol..

[53] Welc N., Owczarek M., Jałowska M., Dańczak-Pazdrowska A. (2025). High-intensity focused ultrasound—Application, effects and complications. Australas. J. Dermatol..

[54] Haykal D., Sattler S., Verner I., Madhumita M., Cartier H. (2025). A systematic review of high-intensity focused ultrasound in skin tightening and body contouring. Aesthetic Surg. J..

[55] Winkie M.J., Sakunchotpanit G., Salazar C.E., Gunasekera N.S., Buzney E.A., Nambudiri V.E. (2024). A focused review of visible light therapies for vitiligo. Photodermatol. Photoimmunol. Photomed..

[56] Thakur V., Bishnoi A., Vinay K., Kumaran S.M., Parsad D. (2021). Vitiligo: Translational research and effective therapeutic strategies. Pigment Cell Melanoma Res..

